# Loci associated with adult stature also affect calf birth survival in cattle

**DOI:** 10.1186/s12863-015-0202-3

**Published:** 2015-05-03

**Authors:** Goutam Sahana, Johanna K Höglund, Bernt Guldbrandtsen, Mogens S Lund

**Affiliations:** Center for Quantitative Genetics and Genomics, Aarhus University, P.O. Box 50, DK-8830 Tjele, Denmark; Present address: Department of Animal Science, Aarhus University, P.O. Box 50, DK-8830 Tjele, Denmark

**Keywords:** QTL, Calving traits, Stature, Cattle

## Abstract

**Background:**

Understanding the underlying pleiotropic relationships among quantitative traits is necessary in order to predict correlated responses to artificial selection. The availability of large-scale next-generation sequence data in cattle has provided an opportunity to examine whether pleiotropy is responsible for overlapping QTL in multiple economic traits. In the present study, we examined QTL affecting cattle stillbirth, calf size, and adult stature located in the same genomic region.

**Results:**

A genome scan using imputed whole genome sequence variants revealed one QTL with large effects on the service sire calving index (SCI), and body conformation index (BCI) at the same location (~39 Mb) on chromosome 6 in Nordic Red cattle. The targeted region was analyzed for SCI and BCI component traits. The QTL peak included LCORL and NCAPG genes, which had been reported to influence fetal growth and adult stature in several species. The QTL exhibited large effects on calf size and stature in Nordic Red cattle. Two deviant haplotypes (HAP1 and HAP2) were resolved which increased calf size at birth, and affected adult body conformation. However, the haplotypes also resulted in increased calving difficulties and calf mortality due to increased calf size at birth. Haplotype locations overlapped, however linkage disequilibrium (LD) between the sites was low, suggesting that two independent mutations were responsible for similar effects. The difference in prevalence between the two haplotypes in Nordic Red subpopulations suggested independent origins in different populations.

**Conclusions:**

Results of our study identified QTL with large effects on body conformation and service sire calving traits on chromosome 6 in cattle. We present robust evidence that variation at the LCORL and NCAPG locus affects calf size at birth and adult stature. We suggest the two deviant haplotypes within the QTL were due to two independent mutations.

**Electronic supplementary material:**

The online version of this article (doi:10.1186/s12863-015-0202-3) contains supplementary material, which is available to authorized users.

## Background

Genetic correlation between two complex (quantitative) traits may be due to pleiotropy, i.e. the same polymorphism affecting both traits, or linkage disequilibrium (LD) between loci affecting individual traits. Genome-wide association studies (GWAS) have identified many genetic loci that harbor variants associated with multiple traits, which highlights the traits sharing common genetic pathways, and emphasizes the relevance of pleiotropy in complex traits [1]. Therefore, understanding the underlying pleiotropic relationships between quantitative traits is integral to predicting correlated responses to artificial selection [2]. The availability of large-scale next-generation sequence data in cattle has provided an opportunity to examine whether pleiotropy is responsible for overlapping QTL in multiple economic traits.

In a genome scan using whole genome sequence variants, we discovered QTL with a large effect on the service sire calving index (SCI), and body conformation index (BCI) at the same location (~39 Mb) on chromosome 6 (BTA6) in a Nordic Red cattle population (Additional file 1). SCI is composed of two traits: calving ease and stillbirth. Calving ease is a characteristic of the cow and calf. Calving outcome is influenced by the cow’s pelvic area, her capacity to prepare to calve, and by the calf size. Service sire calving ease (SCE) measures the tendency of calves from a particular service sire to be born more or less easily, while daughter calving ease (DCE) measures the ability of a particular cow (daughter) to calve easily. Stillbirth (SB) is expressed as calf stillborn birth percentage, including calves born alive but dead within 24 hours of birth. Similar to calving ease, SB might be influenced by genetic contributions from the service sire (sire stillbirth, SSB), and/or genetic components from the cow (daughter stillbirth, DSB).

BCI is a composite index of seven conformation traits (Table 1). Since the 1990s, many countries have used body conformation traits in dairy cattle breeding programs [3]. Although these specific traits are not of economic interest to breeders, the traits themselves are closely related to many economically important traits, including cattle health, productivity, and lifespan [4-7]. Fogh et al. [8] described the joint Nordic model for genetic evaluation for type traits in Denmark, Sweden, and Finland. Stature, one of the component traits exhibited the highest correlation (r = 0.83) with BCI [9]. Stature and gestation length are genetically correlated in cattle (r = 0.49) [7]. Stature is extensively applied as a model for complex trait association studies in humans. Therefore, the genetic architecture of this trait is well studied [10].

**Table 1:** **Number of records for calving and body conformation traits; lead SNPs associated with traits in single marker analyses**

**Trait**	**Trait abbreviation**	**Number of records**	**Highest significant SNP**				
			**rs-ID**	**Position (bp)**	**MAF**	**-log**_**10**_ **(p-value)**	**Effect ± SE**
Stature	ST	3926	rs111018320	38133743	0.40	81.9	4.0 ± 0.2
Body depth	BD	3379	NA	38229392	0.26	26.6	3.2 ± 0.3
Chest width	CW	3379	rs381402811	38196678	0.26	35.7	3.9 ± 0.3
Dairy form	DF	3378	rs43454966	40236966	0.25	11.8	1.8 ± 0.3
Top line	TL	2814	NA	37536932	0.17	6.5	−1.8 ± 0.3
Rump width	RW	3380	NA	39078732	0.22	37.8	3.9 ± 0.3
Rump angle	RA	3380	rs110132087	40335653	0.39	11.3	−1.7 ± 0.3
Service sire calving ease in first lactation	SCEF	4631	rs109301655	38956499	0.08	80.2	−6.4 ± 0.3
Service sire calving ease in later lactations	SCEL	4631	rs109301655	38956499	0.08	83.8	−7.3 ± 0.4
Service sire stillbirth in first lactation	SSBF	4631	NA	38700703	0.22	56.6	−4.2 ± 0.3
Service sire stillbirth in later lactations	SSBL	4631	NA	38700703	0.22	34.7	−3.3 ± 0.3
Calf size in first lactation (service sire effect)	SCSF	4631	rs109301655	38956499	0.08	88.7	6.1 ± 0.3
Calf size in later lactations (service sire effect)	SCSL	4631	rs109301655	38956499	0.08	106.0	6.4 ± 0.3
Service sire calving index	SCI	4631	NA	38700703	0.22	57.9	−4.3 ± 0.3
Body conformation index	BCI	3371	NA	38914033	0.22	80.4	5.0 ± 0.3

rs-ID = reference SNP identification; MAF = minor allele frequency; effect = allele substitution effect for the SNP; SE = SE for the SNP effect; NA = not available.

SCI is associated with prenatal growth, while BCI is influenced by postnatal growth. Therefore, we presume genes associated with both traits affect calf size at birth as well as adult stature. Two genes, LCORL and NCAPG are located at the QTL peak observed in Nordic Red cattle. Eberlein et al. [11] identified a non-synonymous polymorphism in the NCAPG gene resulting in divergent bovine fetal growth. The LCORL and NCAPG genes were suggested as candidate genes for withers height in German Warmblood, and other horse breeds [12,13]. The LCORL gene has also been reported to influence height and fetal growth in humans [14]. Therefore, an examination of whether the same genetic factors (LCORL and NCAPG) underlie the QTL affecting SCI and BCI in Nordic Red cattle is of value.

Fetal growth has an important impact on calving difficulties, because it is highly correlated with dystocia and stillbirth incidence [15]. Therefore, the identification of genes responsible for these traits facilitates our further understanding of the factors and pathways involved in fetal growth, and possible genetic correlations with other undesirable traits in breeding goals, such as stillbirth and dystocia. Knowledge of genetic relationships between early-in-life traits (size at birth), and adult stature could help interpret selection regimes for desired adult stature, while improving calving ease and calf size. Genetic selection can select genes which improve character traits, but do not adversely affect calving ease and calf survival. SCI and BCI are composite traits, and have several sub-traits. Therefore, an association study of the sub-indices might resolve which trait(s) are directly affected by the QTL segregating on bovine chromosome 6 (BTA6). Studies have provided clear evidence that BTA6 is involved in growth and size phenotypes across multiple cattle breeds [16-19]. Therefore, the objectives of this study were as follows: 1) examine whether a common genetic factor on BTA6 is responsible for SCI, BCI, and their component traits; and 2) evaluate whether LCORL and NCAPG, which has been reported to affect fetal growth and adult stature in bovine and several other species are responsible for the QTL effect identified in Nordic Red cattle.

## Methods

A brief overview of the analyses is as follows. Imputation of 50 k genotypes to whole genome sequence (WGS) variants for Nordic Red cattle bulls was done in two steps. First, high density (HD) genotypes were imputed from 50 k genotypes using HD multi-breed reference genotype data. Then the imputed HD genotypes were further imputed to WGS variants using a multi-breed WGS reference. Genome-wide association analysis with the imputed WGS variants was done in two steps: 1) using a linear mixed model without considering the relationship among all individuals except half-sib family (sire-son) relationships and 2) followed by linear mixed model (LMM) analysis within the targeted region considering relationships among all of the bulls. A genome-wide scan with WGS variants using LMM considering the relationships among all individuals is computationally prohibitive. Haplotypes were constructed using top associated markers assuming they all had high linkage disequilibrium with the causal variant and haplotype based association was carried out using LMM analysis.

### Animals and traits

No animal experiments were performed in this study, and, therefore, approval from the ethics committee was not required. The study was conducted in Nordic Red cattle (RDC) from Denmark (RDCDNK), Finland (RDCFIN), and Sweden (RDCSWE). The number of progeny tested bulls with breeding values for traits and genotypes is provided in Table 1. De-regressed breeding values collected as part of routine Nordic Breeding Value Evaluation were used as phenotypes. Details regarding the phenotypes recorded, and models used in routine breeding value prediction can be found at http://www.nordicebv.info.

The indices for linear conformation traits describe different aspects of cow conformation. The linear traits were combined into trait groups describing the overall body form. The body conformation index (**BCI**) is a compound index describing a sire’s genetic potential to produce desirable characteristics for various body measures in progeny. It is predicted as a linear combination of breeding values for stature, body depth, chest width, dairy form, top line, rump width, and rump angle. The definition of conformation traits used in this study was consistent with the International Committee for Animal Recording (ICAR) standards (http://www.icar.org/Documents/Rules%20and%20regulations/Guidelines/Guidelines_2012.pdf). The type traits were scored using a linear scale from 1 – 9 in Denmark, Sweden, and Finland. These records were corrected for calving age, lactation number, interval from calving, and optimum values fixed for each individual trait for each breed. In the joint Nordic evaluation for conformation traits, data from Finland, Sweden and Denmark were included.

Service sire calving index (S**CI**) is a compound index reflecting a sire’s additive genetic effects for calving ease and calf survival in progeny. SCI was predicted by combining service sire breeding values for the following: 1) stillbirth in first (**SSBF**) and later (**SSBL**) lactations; 2) calving ease in first (**SCEF**) and later (**SCEL**) lactations; and 3) calf size in first (**SCSF**) and later (**SCSL**) lactations. We followed Boelling et al. to estimate breeding values for calving traits in Denmark, Sweden, and Finland [20]. The heritabilities for sire calving traits for first lactation in Nordic Red cattle are 0.04 for SSBF, 0.08 for SCEF and 0.20 for SCSF and for later calving the values are 0.01, 0.05 and 0.18 respectively [20].

Accuracies (r) of de-regressed proofs used in this study were high. Average accuracies for DRPs ranged from 0.73 (chest width) to 0.89 (stature) for type traits. Average accuracies ranged from 0.57 (SCSL) to 0.75 (SSBF) for SCI component traits. For example, the DRP accuracy distribution for trait stature is presented in Additional file 1: Figure S1.

### SNP chip and genotyping

Genomic DNA was extracted from whole blood or semen. Bulls were genotyped using the Illumina Bovine SNP50 BeadChip (Illumina Inc., San Diego, CA) version 1 or 2. The SNP selection quality parameters were an 85% minimum call rate for individuals and 95% for loci. Marker loci without a known map position, with minor allele frequencies (MAFs) < 5%, and deviation from Hardy Weinberg proportions (χ^2^-test, 1 *df*, *P* < 0.00001) were excluded. The SNP number after quality control was 43,415 in the 50 k dataset. In addition, a multi-breed reference of 2,036 genotypes (902 Holstein, 735 Nordic Red and 399 Danish Jersey) using the Illumina BovineHD Genotyping BeadChip was available in-house, and from the *EuroGenomics* consortium [21]. The quality control parameters set for HD data were similar, and followed the 50 k chip protocol as described above. The SNP number after quality control for the BovineHD chip was 648,219.

### Whole genome sequencing

Reference sequences used for imputation of Nordic animals were represented by whole genome sequences carried out at Aarhus University [22], and from the 1000 bull genomes project [23]. Nordic bulls were sequenced using Illumina sequencers at Beijing Genomics Institute, Shenzhen, China, and at Aarhus University. Shotgun paired-end sequencing with a 91 or 100 base pair read length was applied. Where necessary, *fastq* data were converted from Illumina to Sanger quality encoding using a patched version of *maq* [24]. Data were aligned to the cattle genome UMD3.1 assembly [25] using *bwa* [26]. Aligned sequences were converted to raw BAM files with *samtools* [27]. Sequences were realigned around indels using the Genome Analysis Toolkit [28]. Quality scores were re-calibrated using the Genome Analysis Toolkit following the Human 1000 Genome [29]. The Genome Analysis Toolkit’s *UnifiedGenotyper* was employed to call variants. Genomes for the 1000 bull genomes project were sequenced in a number of laboratories, and collected at the Department of Primary Industries, Victoria, Australia. Sequences were subsequently aligned to the same reference genome established at Aarhus University. Variant calling was conducted using samtools’s *mpileup* function. Variant Call Files from Aarhus University, and the 1000 bull genomes project were combined with the Genome Analysis Toolkit’s *CombineVariants.*

### Imputation to high-density SNP arrays and full sequence levels

Markers on the 50 k chip, which were not included on the HD chip, were excluded as reference markers. The SNP genome positions were obtained from the UMD3.1 Bovine genome assembly [25]. Imputation of the 50 K SNP data to the full bovine sequence was conducted in two steps. First, 50 K genotypes for 12,322 Nordic bulls were imputed to HD genotypes using IMPUTE2 software [30]. In the second imputation step, the 12,322 bulls imputed to HD genotypes were imputed to the full sequence level. The whole genome sequences from 242 dairy cattle were used as reference sequences to estimate the imputed HD data to the whole genome level using BEAGLE software [31]. HD imputation to sequence data was completed by dividing chromosomes into chunks of 20,000 consecutive markers with an overlap of 250 markers at each end to minimize imputation error at chunk ends.

The 242 dairy cattle whole genome sequences were from Aarhus University, and the 1000 bull genomes project. The reference included 132 Holstein, 42 Jersey, 52 Nordic Red, and 16 Brown-Swiss bulls [32]. Only polymorphisms identified from the reference dataset were imputed. The insertion-deletion (INDEL) was deleted for positions containing a SNP, and an INDEL polymorphism. SNPs at positions exhibiting a lack of congruency between alleles from sequence and HD data were deleted. The reference data were pre-phased with BEAGLE v3.3.2 [31]. Markers with an imputation certainty (R^2^) < 0.90 were removed. The total marker number for chromosomes 1–29 was 8,938,927. The imputation accuracy for this data was reported earlier [32].

### Statistical methods for association analysis

A genome scan was conducted for BCI and SCI using a sire model without considering relationships among individuals other than between sire and son. The model is described in the Additional file 1. Based on results, a region harboring a QTL for BCI and SCI was targeted for single marker and haplotype-based analyses using a linear mixed model (LMM) [33]. Complex familial relationships are the primary confounding factor in livestock population genome-wide association studies (GWAS). However a LMM, which includes relationships among individuals through polygenic effects, can control false positive detection rates of associations due to family structure and population stratification in cattle [34,35]. Bonferroni multiple testing correction was applied for 8,938,927 simultaneous tests (−log_10_ (*P*-value) = 8.25).

### Linear mixed model analysis

The following linear mixed model (LMM) was applied for association analyses of the BTA6 targeted region for the two indices (BCI and SCI), and the component traits (Table 1). The associations between SNPs and phenotypes were assessed by a single SNP regression analysis for each SNP separately. The model is as follows:

$$ {y}_j=\mu +b{x}_j+{u}_j+{p}_k+{e}_j $$

where y_j_ is the phenotype (de-regressed EBV) for individual *j*, *μ* is the overall mean, *b* is the allelic substitution effect, *x*_*j*_ is the number of copies of an allele (with arbitrary labeling) of the SNP count in individual *j* (corresponding to 0, 1, or 2 copies), *u*_*j*_ are random polygenic effects, *р*_*k*_ is the effect of the k-th population (*k* = 1, 2 or 3), and *e*_*i*_ is the random environmental deviate for individual *j*. The vector ***u*** *= {u*_*j*_*}* follows a multivariate normal distribution $\boldsymbol{u}\sim \mathrm{N}\left(\mathbf{0},\boldsymbol{A}\ {\sigma}_u^2\right)$ , where ***A*** is the additive relationship matrix, and ${\sigma}_u^2$ is the polygenic variance. The vector of random environmental deviates ***e =*** *{e*_*j*_*}* follows a multivariate normal distribution $N\left(\mathbf{0},{\boldsymbol{W}}^{-1}{\sigma}_e^2\right)$, where ${\sigma}_e^2$ is the error variance, and ***W*** is the diagonal matrix containing weights of de-regressed estimated breeding values. The weight *w*_*j*_ for individual *j* is estimated as ${w}_j={r}_j^2/\left(1-{r}_j^2\right)$, where *r*_*j*_^*2*^ is the reliability of the de-regressed EBV of individual *j.* Values of ${r}_j^2>0.98$ were reduced to 0.98 to avoid excessively large sire weights under large numbers of progeny records. The model was fitted by REML using DMU software [36]. DMU was used to obtain fixed effects estimates and standard errors. Testing for marker effect was performed using a *t*-test against a null hypothesis of *H*_*0*_*: b = 0,* and the same Bonferroni multiple testing corrected significance threshold (−log_10_ (*P*-value) = 8.25) was used as in the genome scan (Additional file 1).

### Random haplotype model (RHM)

Haplotypes were constructed for markers selected from LMM analyses using BEAGLE software [31]. We used a linear mixed effect model with random polygenic and haplotype effects following Boleckova et al. [37] as follows:

$$ {y}_j=\mu +{q}_{h1j}+{q}_{h2j}+{u}_j+{p}_k+{e}_j $$

where, *q*_*h1j*_ and *q*_*h2j*_ are random effects of the two haplotypes carried by the j-th individual. Each haplotype effect is assumed independent and identically distributed (i.i.d), with a normal prior distribution ${q}_{hij}\sim N\left(0,{\upsigma}_h^2\right)$, where ${\upsigma}_h^2$ is variance explained by the haplotype. The other terms in the RHM are as described for LMM. Note the haplotype effects are the same regardless of paternal or maternal origins, corresponding to an assumption of absence of parental imprinting. The significance of the haplotype substitution effect was assessed by a likelihood ratio test, comparing the RHM model with a null-model composed of mean, polygenic and breed effects, and random error terms, but haplotype effects were not included. The likelihood ratio test statistic has a χ^2^ distribution with 1 *df*. The analysis was performed using the DMU software package [36].

Tests were conducted to examine if a significant candidate SNP or haplotype alone was sufficient to explain the variation resulting from a QTL region. Analyses were repeated with incorporation of the candidate SNP or haplotype as a fixed effect. If other SNPs or haplotypes remained significant in the presence of the new systematic effect, the original candidate alone was not sufficient to account for the entire effect of the QTL region.

## Results

### Genome scan for service sire calving index (SCI) and body conformation index (BCI) using a sire model

**Figure 1:**
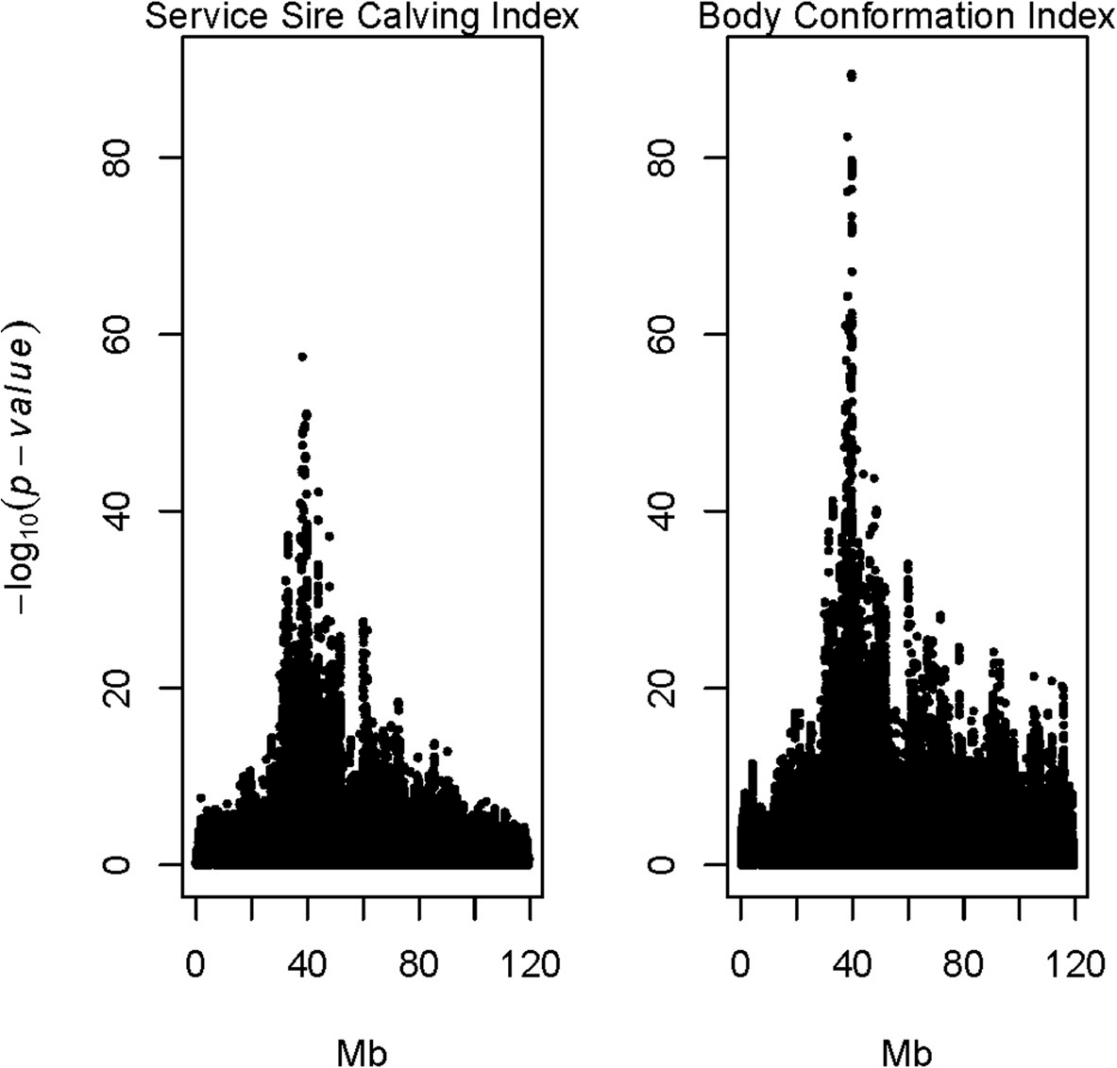
Association analysis using a sire model for SCI and BCI for chromosome 6 in Nordic Red cattle.

The genome-wide associations scan using the sire model (Additional file 1) for SCI detected one QTL with large effect located on BTA6 (−log_10_ (*P*-value) = 57.5, Figure 1, Additional file 1: Table S1). The most significant SNP was located at 38,127,504 bp, and had a minor allele frequency (MAF) of 0.31. The 20 most significant SNPs associated with this QTL are presented in Additional file 1: Table S1. The SNPs were located between 38,127,504 and 39,750,084 bp. Markers within this peak QTL region showed no significant association with the daughter calving index (details not presented). Therefore, results indicated this QTL influenced SCI, but had no effect on the daughter calving index.

The same sire-model was used to scan for QTL affecting BCI. The largest QTL affecting BCI on BTA6 was located at 39,685,188 bp, with a MAF of 0.40 and the –log_10_ (*P*-value) was 89.4 (Figure 1, Additional file 1: Table S2). The 20 most significant SNPs associated with the conformation QTL are presented in Additional file 1: Table S2, and were located between 38,127,504 and 39,697,560 bp.

The most significant SNPs for calving and conformation indices were 1,557,684 bp apart; however the QTL peaks overlapped (Additional file 1: Tables S1 and S2). Therefore, we further examined if the overlapping QTL for calving and conformation were due to pleiotropic effects of the same gene or two linked genes, each effecting one index, or multiple factors effecting one or both traits.

### Association study with Linear Mixed Models for the targeted region on chromosome 6

The Linear Mixed Model (LMM) analyses for conformation and calving indices and their component traits revealed overlapping peaks in the targeted region on BTA6 (~37.5-40.5 Mb). The most significant associated SNPs from LMM for calving, conformation and the component traits are presented in Table 1. The most significant SNP for SCI was located at 38,700,703 bp (−log_10_ (*P*-value) = 57.9). This SNP was also the most significantly associated SNP for calf survival, i.e. stillbirth in first (**SSBF**) and later (**SSBL**) lactations. The SNP located at 38,956,499 bp showed the most significant association with calf size and calving ease. The allele substitution effect of this SNP on calf size and calving ease exhibited an inverse association, i.e. the allele increasing calf size at birth increased calving difficulty. The SNP most significantly associated with body conformation was at 38,914,033 bp (−log_10_ (*P*-value) = 80.4). It was located 213,330 bp from the SNP most associated with SCI. The SNP most significantly associated with stature was at 38.13 Mb (−log_10_ (*P*-value) = 81.9). However, a number of SNPs around 39.77 Mb showed a very strong association with stature (−log_10_ (*P*-value) = 80.6).

**Figure 2:**
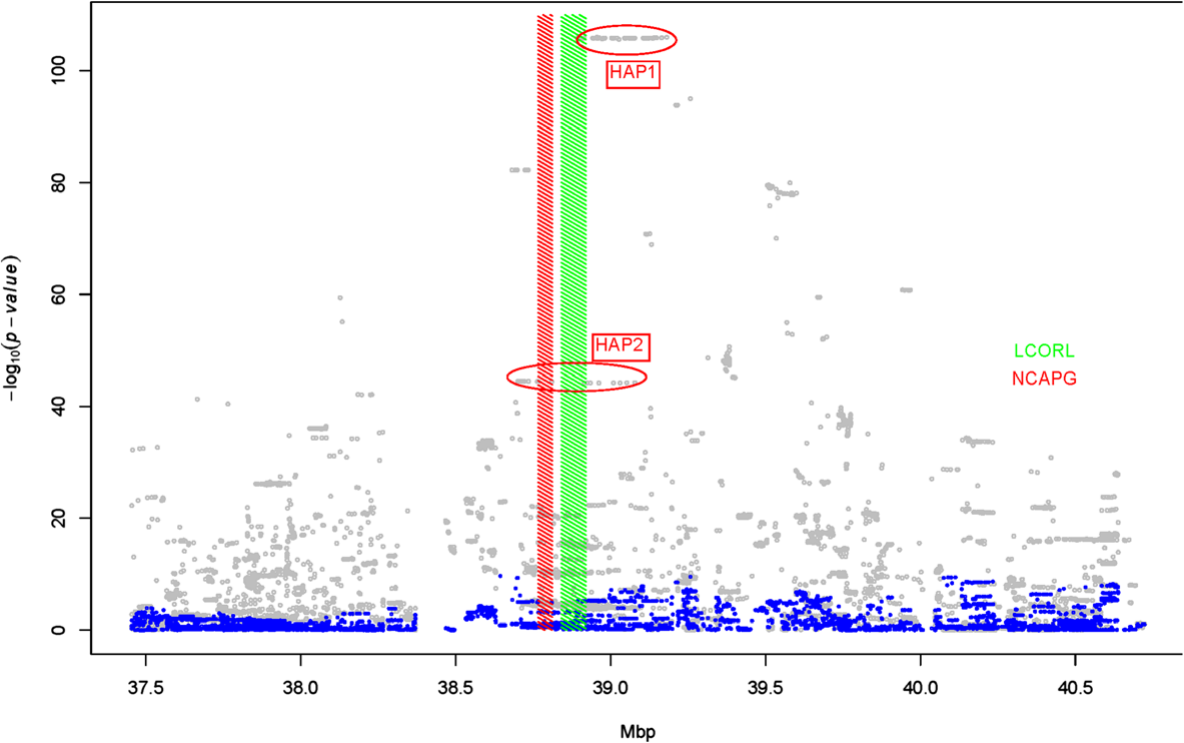
Single marker association analysis for calf size in later lactations using a linear mixed model (LMM, grey dots). Two haplotypes were constructed at the QTL peak and the blue dots are when two haplotypes were fitted as covariates in the model. HAP1 and HAP2 are two haplotype classes from the two haplotype regions showing the effect on the phenotype. Red and green rectangles indicate NCAPG and LCORL gene locations.

**Figure 3:**
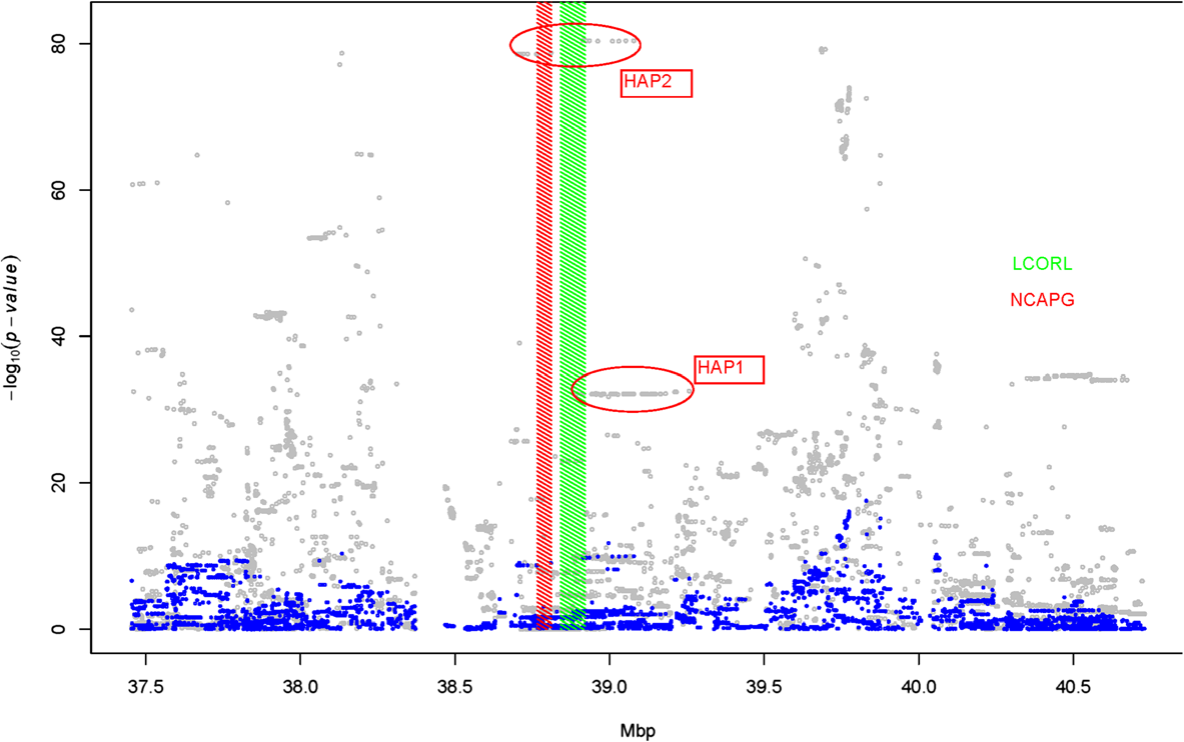
Single marker association analysis for the targeted BCI region using a linear mixed model (LMM, Grey dots). Two haplotypes were constructed at the QTL peak and the blue dots are when two haplotypes were fitted as covariates in the model. HAP1 and HAP2 are two haplotype classes from the two haplotype regions showing the effect on the phenotype. Red and green rectangles indicate NCAPG and LCORL gene locations.

**Figure 4:**
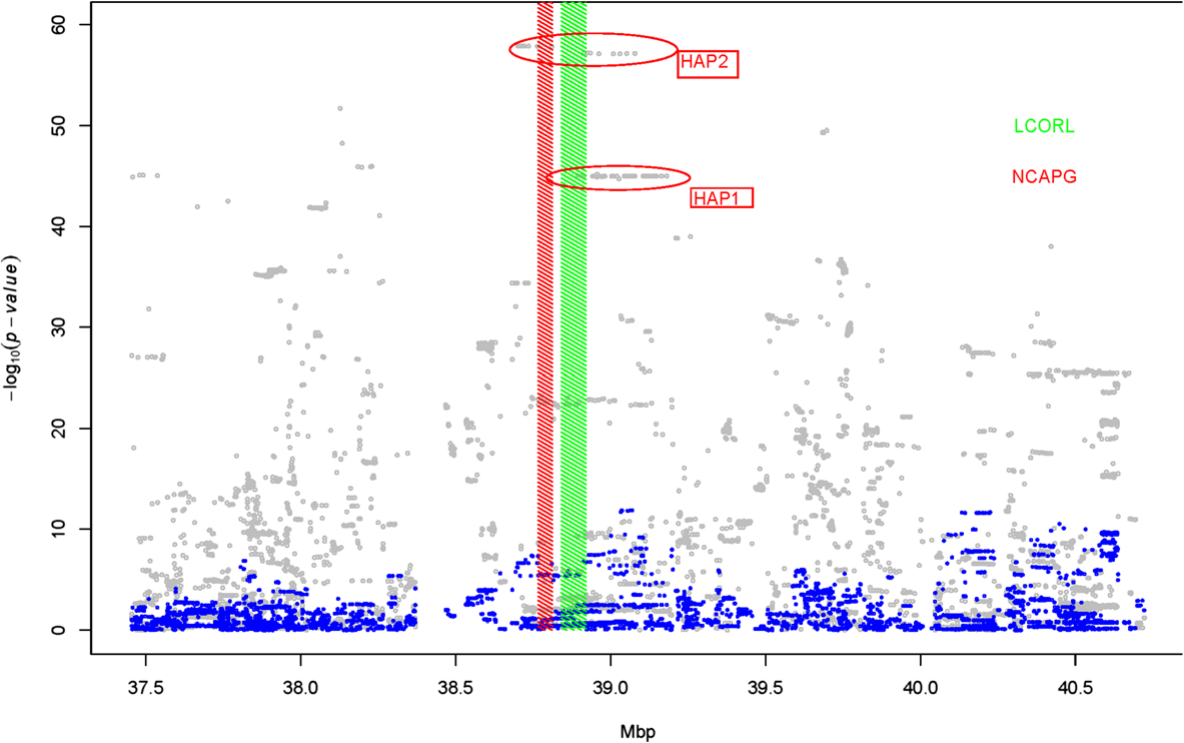
Single marker association analysis for the targeted SCI region using a linear mixed model (LMM, grey dots). Two haplotypes were constructed at the QTL peak and the blue dots are when two haplotypes were fitted as covariates in the model. HAP1 and HAP2 are two haplotype classes from the two haplotype regions showing the effect on the phenotype. Red and green rectangles indicate NCAPG and LCORL gene locations.

Association analyses using LMM for targeted regions indicated the possible presence of multiple factors affecting conformation and calving index traits. First, results showed SNPs with the largest effects for calf size and calving ease had a MAF of 0.08 compared to 0.22 for SNPs with large effects on SCI, BCI, and stillbirths. Second, association plot results showed different markers at the peak for different traits (Figures 2, 3 and 4). For example, calf size in later lactations (SCSL) indicated a number of markers with similar –log_10_ (*P*-values) at ~106 (Figure 2), while BCI showed another marker set with –log_10_ (*P*-values) at ~80 (Figure 3). However, these two marker group locations partly overlapped. It was evident from the association plots that the peak QTL region was covered by long stretches of markers showing similar associations with the phenotypes (Figures 2, 3 and 4).

### Haplotype-based association analysis

Two genomic regions were selected for haplotype based association analyses. The highest –log_10_ (*P*-value) for the targeted region among all traits analyzed was observed for SCSL (Figure 2). The positions for markers most significantly associated with SCSL overlapped with the LCORL gene, which was previously reported to have an effect on horse [12,13] and human fetal growth and/or body size [14]. The 15 markers with the largest effects on SCSL were located within the LCORL gene (Additional file 1: Table S3). These markers were used to construct the haplotype for haplotype-based association analysis with random haplotype model (RHM). Among the haplotype classes within the LCORL gene, the haplotype marked as HAP1 (Figures 2, 3 and 4) had the biggest effect on calving ease in first (**SCEF**) and later (**SCEL**) lactations, and calf size in first (**SCSF**) and later (**SCSL**) lactations. The second haplotype was constructed using the most significant markers for BCI (Figure 3, Additional file 1: Table S4). Among the haplotype classes from this region, the haplotype labeled as HAP2 (Figures 2, 3 and 4) had biggest effect on SCI, stillbirth in first (**SSBF**) and in later (**SSBL**) lactations, and body-depth. These two haplotypes were fitted individually and jointly in the RHM to determine if one or both haplotypes were sufficient to explain the QTL variance observed in the targeted region for the traits analyzed.

#### Hap1

**Figure 5:**
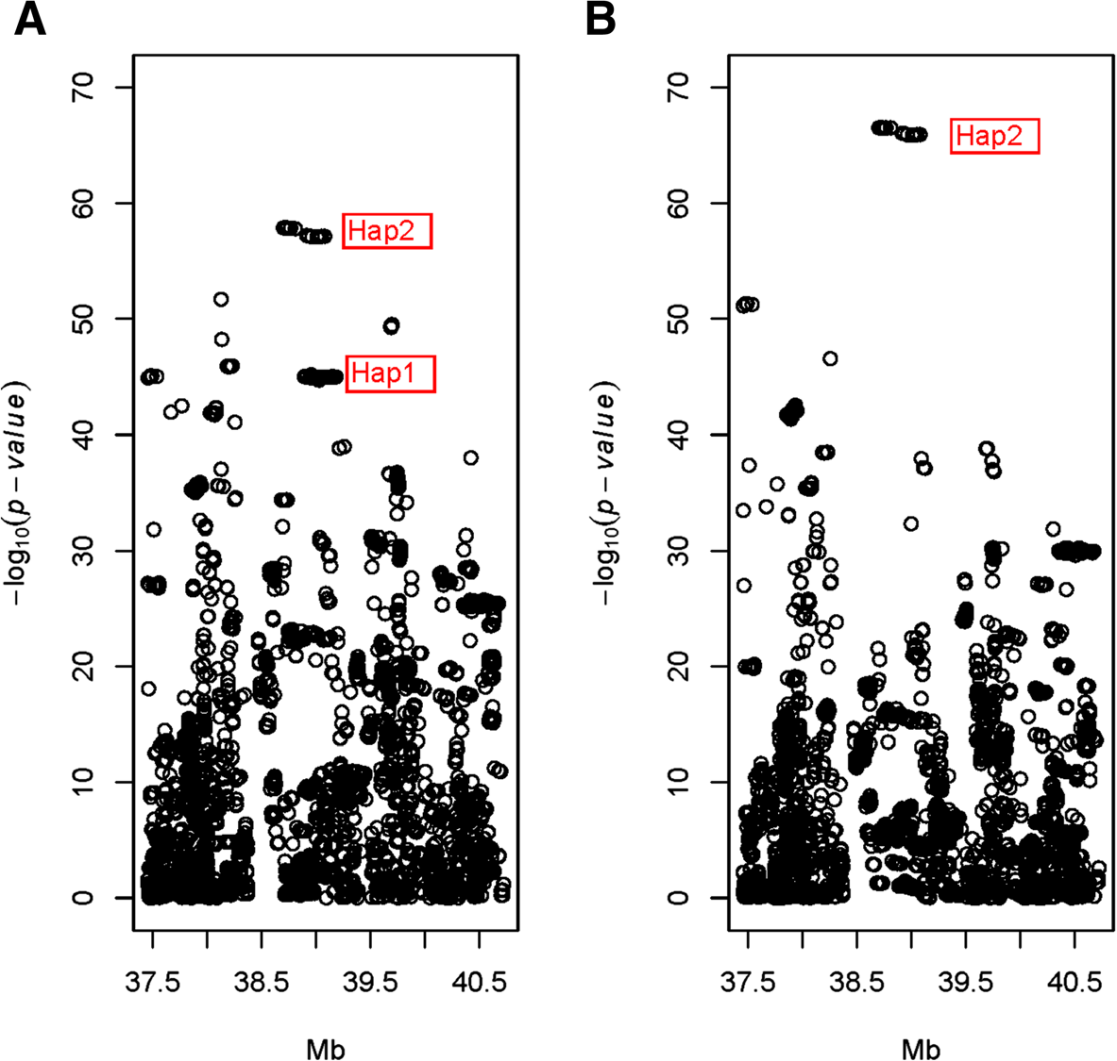
Single marker analysis using liner mixed model (LMM) for SCI. **A)** single marker analysis, **B)** single marker analysis with haplotype from LCORL gene as cofactor in the model. HAP1 and HAP2 are two haplotype classes from two haplotype regions having the biggest effect on phenotypes.

Single marker analysis revealed the strongest SCSL association signal at ~38,893,987 to ~39,165,804 bp with –log_10_ (*P*) = 106.9 (Figure 2). The first step of the RHM analysis included the LCORL gene (HAP1) haplotype as a fixed effect. The region was scanned to examine whether HAP1 explained all of the QTL variance for the two indices (SCI and BCI) and the sub-indices (described in Method section). There were only two haplotype labels segregating ‘CAACCAGCCCGCAAG’ and ‘CTCTTTCATTTAC_CA’ with frequencies of 0.915 and 0.085, respectively in the combined Nordic Red cattle from three countries. However, relative to the three Nordic Red breeds, the haplotype with reduced frequency was much higher in RDCDNK (0.420) compared to RDCFIN (0.003), RDCSWE (0.019), RDCSWE and RDCFIN which showed absence of homozygosity for this haplotype. Additive haplotype effects for all the traits are shown in Table 2. Average SCSL breeding values were higher in RDCDNK (107.2 ± 0.52) compared with RDCFIN (99.1 ± 0.12) and RDCSWE (98.6 ± 0.14). Average SCSF breeding values were respectively 106.1 ± 0.54, 99.1 ± 0.13 and 98.3 ± 0.15 for RDCDNK, RDCFIN and RDCSWE. A significantly higher calf size in RDCDNK could be due to a high haplotype frequency responsible for increased calf size. However, fitting this haplotype in the model, and scanning the region showed the haplotype alone did not explain all QTL variance (Figure 5A-B).

**Table 2:** **Estimates of two haplotype effects fitted individually or jointly in the model**

**Trait**	**M1 = breed + HAP1 + Animal**		**M2 = breed + HAP2 + Animal**		**M3 = breed + HAP1 + HAP2 + Animal**			
	**Eff. HAP1**	**SE. HAP1**	**Eff. HAP2**	**SE. HAP2**	**Eff. HAP1**	**SE. HAP1**	**Eff. HAP2**	**SE. HAP2**
Stature	5.09	0.40	3.84	0.22	5.89	0.39	4.10	0.22
Body depth	2.69	0.49	2.84	0.29	3.20	0.49	3.00	0.29
Chest width	3.64	0.51	3.36	0.31	4.32	0.51	3.59	0.31
Dairy form	0.61	0.43	0.48	0.26	0.69	0.43	0.52	0.26
Rump width	2.88	0.45	3.52	0.28	3.49	0.45	3.72	0.28
Rump angle	−0.23	0.44	−0.62	0.27	−0.26	0.45	−0.64	0.27
SCEF	−5.97	0.32	−2.6	0.16	−6.54	0.31	−2.84	0.15
SCEL	−6.85	0.36	−2.75	0.18	−7.46	0.35	−3.03	0.17
SSBF	−5.86	0.48	−3.66	0.24	−6.58	0.47	−3.90	0.24
SSBL	−5.35	0.47	−2.90	0.24	−5.96	0.46	−3.12	0.24
SCSF	5.74	0.29	2.07	0.14	6.23	0.28	2.30	0.14
SCSL	6.05	0.28	1.88	0.14	6.51	0.27	2.12	0.13
SCI	−6.78	0.49	−3.78	0.25	−7.54	0.48	−4.05	0.24
BCI	4.79	0.41	4.36	0.24	5.66	0.39	4.65	0.24

SNP from the LCORL gene exhibiting the most significant association with calf size were used for constructing HAP1. SNPs indicating the most significant association with body conformation were used to construct HAP2.

#### Hap2

Twenty-five SNPs were used to construct the HAP2 region (Additional file 1: Table S4). Two haplotype labels were observed for HAP2, with frequencies of 0.76 and 0.24 across three red breeds, and allelic combinations of ‘TTTTATAATCATAGAATAACACTGT’ and ‘CCCCGCGGGTGCGACGAGGTGTCAC’, respectively. Respective frequencies were 0.04, 0.32, and 0.211 in RDCDNK, RDCFIN, and RDCSWE for the reduced frequency haplotype.

#### *Hap1* and *Hap2*

HAP1 and HAP2 were fitted as fixed effects in the mixed model to determine variance explained by these two haplotypes. A significant association for BCI (Figure 3) and SCI (Figure 4) were not detected for any SNPs in the region. The effect size estimates for these two haplotypes are presented in Table 2, and model comparisons in Additional file 1: Table S5. The model comparisons (i.e. none, one, or both haplotypes were fit) where both haplotypes were included exhibited the best fit for all traits based on the Akaike Information Criterion (AIC) [38]. HAP1 and HAP2 increased the breeding values for BIC, calf size, stature, body depth, and chest and rump width, but decreased the breeding values for calving index, calf survival, and calving ease. The squared correlation (*r*^*2*^) between the two haplotype doses was 0.064, indicating very low LD between the two haplotype blocks.

## Discussion

In the present study, we reported QTL with a large effect on calf size and stature in Nordic Red cattle. We identified two deviant haplotypes that increased calf size at birth, and altered adult body conformation. However, the haplotypes also increased calving difficulties, and subsequently calf mortality due to the increase in calf size. Results indicated the haplotype locations overlapped. However, the LD between sites was low, suggesting two independent mutations responsible for similar effects. Several studies reported QTL in the same genomic region on chromosome 6 impacting birth weight, growth, and carcass traits in beef breeds and crossbreds [16-19]. In dairy breeds, studies showed the QTL resulted in stillbirth and dystocia [39-43]. Schrooten et al. [44] mapped the QTL for calving ease at a similar location on BTA6 in Holstein cattle. However, many QTL mapping and association studies in Holstein for calving traits did not identify QTL segregating on this region on chromosome 6 [45-48].

The cattle population we used for association analysis had both population structure (three sub-populations) and family relationship (large half-sib families). Therefore a linear model including the full relationship matrix is necessary to avoid false positive associations [33,34]. However, linear model analyses with full relationship matrix for whole genome sequence variants are computational prohibitive. Therefore, association analyses were carried out in two-steps. First, a linear mixed model that considered the sire-son relationship was run, followed by a LMM with full relationship for the variants within the targeted region.

### Low haplotype diversity at the QTL peak

We observed reduced haplotype diversity at the QTL peak compared to the rest of the chromosome (Figure 2). One of the reasons could be that a small reference population was used to impute the sequence variants. There were 242 animals in the reference population, only 56 animals were from Nordic Red Cattle [32]. Therefore, the haplotypes not observed in the reference population could not be imputed. However, in that case we should observe the similar reduction in haplotype diversity across a whole chromosome. A subset of these animals overlapped with the 1000 bull genomes project [49] and a total of 28.3 million sequence variants were observed from 234 sequenced bulls. The average number of heterozygous sites per individual in the 1000 bull genomes project was 1.44 per kilobase, which is higher than has been found in humans [23]. The QTL identified in this study had large effects, and, therefore, strong artificial selection in Nordic red cattle might have increased the frequency of the haplotypes with favorable effects. This was possibly the reason we observed reduced haplotype diversity at the QTL peak.

### Candidate genes underlying the QTL

Soranzo et al. [49] studied human chromosome 4 in relationship to human height, and observed that SNPs from the LCORL and NCAPG genes comprised a linkage disequilibrium block. Sovio et al. [50] showed SNP associations located in this region (LCORL and NCAPG) with trunk and hip axis lengths. It was hypothesized that arginine metabolism was linked to the role of LCORL and NCAPG in growth [51,52]. LCORL gene variants might increase or decrease regulation of genes involved in growth and appetite pathways [53].

The NCAPG and LCORL loci have been investigated in beef cattle populations for effects on carcass traits and body weight. A missense variant (rs109570900) at 38,777,311 bp within NCAPG, which induced a Ile-442-Met substitution in amino acid sequence was associated with carcass weight, the longissimus muscle area, and subcutaneous fat thickness in Japanese Black and Brown cattle [54]. The same variant was significantly associated with fetal growth in Charolais-Holstein crossbreds [11]. Additional cattle studies discovered a major QTL affecting fetal growth, and related traits at the same location on BTA6. Casas et al. [16], Kühn et al. [40] and Kneeland et al. [17] mapped a QTL for birth weight and body length on BTA6, and Snelling et al. [55], Gutiérrez-Gil et al. [18] and Weikard et al. [51] provided further evidence that the NCAPG gene was involved in birth and body weight regulation. However, it is not possible to confirm that the same variant was responsible across several species, as the traits were defined differently, and the associated LD haplotype blocks contained several genes. However, strong evidence suggested the NCAPG and LCORL gene region had a large effect on growth related traits across several species, supporting the hypothesis that a conserved locus across species might be responsible for both fetal and adult stature.

### Two haplotypes possibly represent two distinct mutations

We observed two haplotypes within overlapping regions on BTA6, which affected SCI and BCI in Nordic Red cattle. Correlation between imputed haplotype doses for the two haplotype blocks was low, indicating low LDs. Haplotype distinctness was also supported by the model fit. The best model fit was obtained when HAP1 and HAP2 were included in the model. Marked frequency differences were observed in HAP1 and HAP2 from the three country RDC populations. HAP1 was common in RDCDNK (frequency = 0.42), but rare in RDCFIN (0.003) and RDCSWE (0.019). In contrast, HAP2 was rare in RDCDNK (frequency = 0.04), but common in RDCFIN (0.32) and RDCSWE (0.21). We checked the functional annotations for markers included in HAP1 and HAP2. All 15 markers from the LCORL gene, which form HAP1 were intronic variants (Additional file 1: Table S3). The markers in HAP2 were intergenic, intronic, or upstream gene variants (Additional file 1: Table S4). However, the functional annotations from the most informative associated markers were not useful in identifying the responsible polymorphisms. We searched for functionally important variants located within the two haplotypes, which also segregated in Nordic Red cattle (Table 3). Missense variant frequency within the NCAPG gene (rs109570900) at 38,777,311 bp closely matched HAP1 frequencies (Table 3) for the three RDC breeds. The correlation between the missense variant dose and HAP1 was *r* = 0.62 in RDCDNK. This NCAPG gene missense variant was earlier reported from several cattle populations, e.g. the variant exhibited the strongest effect on birth weight in Holstein × Charolais [11], and was associated with carcass weight, longissimus muscle area, and subcutaneous fat thickness in Japanese Black and Brown cattle [54]. Therefore, rs109570900 is a strong candidate for the responsible variant in HAP1. However, without further evidence, it is not possible to conclude whether this variant is the factor underlying HAP1, or the variants showing high association but not annotated yet. It is also possible that the causal polymorphism(s) was not in the analyzed panel of SNPs because they were removed in the quality checking or they are structural variants such as CNVs which were not considered in this study.

**Table 3:** **Functionally important variants located within LCORL gene haplotype on chromosome 6 segregating in Nordic Red cattle; RDCDNK, RDCSWE, and RDCFIN are Nordic Red cattle from Denmark, Sweden, and Finland, respectively**

**Position (bp)**	**Annotation**	**Allele frequency in RDCDNK**	**Allele frequency in RDCSWE + RDCFIN**
38776669	Missense variant; SIFT^1^: tolerated(1); GERP^2^ = −5.05	0 = 0.967; 1 = 0.033	0 = 0.868; 1 = 0.132
38777311	Missense variant; SIFT: deleterious(0.02); GERP = 3.13	0 = 0.633; 1 = 0.367	0 = 0.971; 1 = 0.029
38779781	Missense variant; SIFT: deleterious(0.02); GERP = 4.31	0 = 1.000	0 = 0.941; 1 = 0.059
38808241	Missense variant; SIFT: deleterious(0.04); GERP = 0.886	0 = 0.522; 1 = 0.478	0 = 0.971; 1 = 0.029
38844538	Missense variant; SIFT: tolerated(1); GERP = 2.51	0 = 0.000; 1 = 1.000	0 = 0.985; 1 = 0.015
38844605	Frameshift variant & splice region variant & feature truncation	0 = 0.000; 1 = 1.000	0 = 0.000; 1 = 1.000
38846294	Splice donor variant; GERP = 4.4	0 = 0.943; 1 = 0.057	0 = 0.926; 1 = 0.074
38991938	Inframe insertion	0 = 1.000	0 = 0.865; 1 = 0.135

^1^SIFT = *Sorting Intolerant From Tolerant predicted score* [56]; ^2^GERP = *Genomic Evolutionary Rate Profiling predicted score* [57].

We identified two deviant haplotypes (HAP1 and HAP2) with similar effects on BCI and SCI. The two haplotype locations overlap, but LD between the sites was low. HAP1 frequency was common in RDCDNK, but rare in RDCFIN and RDCSWE, while HAP2 showed reverse frequencies, i.e. common in RDCFIN and RDCSWE, but rare in RDCDNK. Principal component analysis provided clear evidence RDCDNK was genetically divergent from RDCFIN and RDCSWE (results not shown). Therefore, the two haplotypes might have had independent origins in different populations. If the two haplotypes represent two distinct mutations on the same gene, then this would provide an explanation why they had the same effect on calf size at birth and adult stature.

### Trends in stature and calving traits

Opposite trends for calving ease and stature had been reported during the last three decades. For example, the average breeding value (PTA) for daughter calving ease in US Holstein cattle have decreased from 9.3 in 1980 to 6.6 in 2009, while PTA for daughter stillbirths has decreased from 10.6 to 7.2 for the same period (https://www.cdcb.us/eval/summary/trend.cfm?R_Menu=HO.sb#StartBody eval/summary/trend.cfm?R_Menu = HO.sb#StartBody, accessed 30 June 2014). In contrast, first lactation stature in Canadian Holstein has demonstrated steady genetic gain; average stature has increased from < 143 cm in 1991 to 147 cm in 2001 (www.cdn.ca/document.php?id=15, accessed 30 June 2014). The haplotypes identified in Nordic Red cattle increased adult body conformation. The present BCI breeding goal in Nordic Red cattle is for an optimum; however, the genetic trend for stature over time has been positive [8]. This might be due to low weight on body conformation in the breeding goal and/or farmers’ preference for larger cows as the larger animals have a tendency for increased yield. However, large cows tend to have lower fertility, and higher disease incidence [8]. Larger birth weight/size might subsequently result in larger adult size. Selection for increased stature might therefore lead to higher birth weight, and more calving difficulties, which in turn results in higher calf mortality [8]. In this study, we observed that HAP1 and HAP2 also increased calving difficulties and calf mortality. The haplotype information revealed in this study shows promise in practical breeding applications to avoid and prevent negatively correlated responses. However, it remains to be investigated how to incorporate the haplotype information in practical breeding programs.

## Conclusions

We identified several significant markers for body conformation, and service sire calving traits. We presented strong evidence that variation at the LCORL and NCAPG loci affected calf size and adult stature. Our results are congruent with many other studies in cattle, and across other species. However, confirmation of the factor(s) responsible for the mutation(s) requires further study.

### Availability of data

All DNA sequences used were taken from a publicly available assembly. The assembly is available for download (ftp://ftp.ensembl.org/pub/release-73/fasta/bos_taurus/dna). All variations used in the mapping study have been submitted by the 1000 bull genomes project for inclusion in dbSNP (http://www.ncbi.nlm.nih.gov/SNP). Whole genome sequence data for the 234 individuals included in run2 of the 1000 bull genomes project are available at NCBI using SRA no. SRP039339 (http://www.ncbi.nlm.nih.gov/bioproject/PRJNA238491). All annotations were obtained from a publicly available source (http://www.ensembl.org) downloadable including through Variant Effect Predictor (http://www.ensembl.org/info/docs/tools/vep/script/index.html).

## Additional file

Additional file 1:
**Supplementary text, Figure S1 and Table S1-S5.**

## Abbreviations

BTA: *Bos taurus* autosome
CE: Calving ease
CS: Calf size
DCE: Daughter calving ease
DPR: De-regressed proof
HAP: Haplotype
QTL: Quantitative trait loci
RDC: Nordic Red cattle
SB: Stillbirth
SCE: Service sire calving ease
