# Supplementary material for: Loci associated with adult stature also affect calf birth survival in cattle
Source: BMC Genet. 2015 May 3;16:47. doi: 10.1186/s12863-015-0202-3 (PMC4426170; doi:10.1186/s12863-015-0202-3)
Supplement: Additional file 1: — Supplementary text, Figure S1 and Table S1-S5. [file 12863_2015_202_MOESM1_ESM.pdf]

Loci associated with adult stature also affect calf birth survival in cattle

Goutam Sahana, Johanna K. Höglund, Bernt Guldbrandtsen and Mogens S. Lund

Supplementary text

Sire Model (SM) for Genome Scan. The genome was initially scanned using two index traits; service sire calving index (SCI) and body conformation index (BCI), following the sire model. A single SNP analysis was conducted, where each SNP was consecutively tested for phenotype association. The following model was used to estimate SNP effects:

$$y_{ij} = \mu + bx_{ij} + s_i + p_k + e_{ij}$$

where y_{ij} is the de-regressed breeding value of individual *j*, belonging to the half-sib (sire) family *i*, μ is the general mean, *b* is the allelic substitution effect, x_{ij} is the number of allele copies (with arbitrary labeling) of SNP count in individual *j* (corresponding to 0, 1, or 2 copies), and s_i is the random effect of the *i*-th half-sib family assumed to exhibit a normal distribution $s \sim N(0,\sigma_s^2)$, where σ_s^2 is the sire variance, p_k is the effect of the k-th population. The vector of random environmental deviates $\boldsymbol{e}=\{e_j\}$ follows a multivariate normal distribution $N(\mathbf{0}, \boldsymbol{W}^{-1}\,\sigma_e^2)$, where σ_e^2 is the error variance, and $\boldsymbol{W}$ is the diagonal matrix containing weights of de-regressed estimated breeding values. The weight w_j for individual *j* is estimated as $w_j = r_j^2/(1-r_j^2)$, where r_j^2 is the reliability of the de-regressed EBV of individual *j*. Values of $r_j^2 > 0.98$ were reduced to 0.98 to avoid excessively large sire weights under large numbers of progeny records. All statistical analyses were conducted using DMU software (Madsen & Jensen, 1910). The null hypothesis H_0: b = 0 was tested using a *t*-test. A significant association between a SNP and trait were accepted if the $-\log_{10}$ (*P*-value) was > 8.25 (applying a Bonferroni multiple test correction for

simultaneous testing of 8,938,927 SNPs). Pair-wise LD between top associated markers was estimated using PLINK software (Purcell et al. 2007).

Reference

Madsen P, Jensen J: DMU A Package for Analysing Multivariate Mixed Models. 6 edn; 2013.

Purcell S, Neale B, Todd-Brown K, Thomas L, Ferreira MA, Bender D, Maller J, Sklar P, de Bakker PI, Daly MJ, Sham PC. PLINK: A tool set for whole-genome association and population-based linkage analyses. Am. J. Hum. Genet. 2007; 81: 559–575

Figure S1 Distribution of accuracies for de-regressed proofs for stature in Nordic Red cattle.

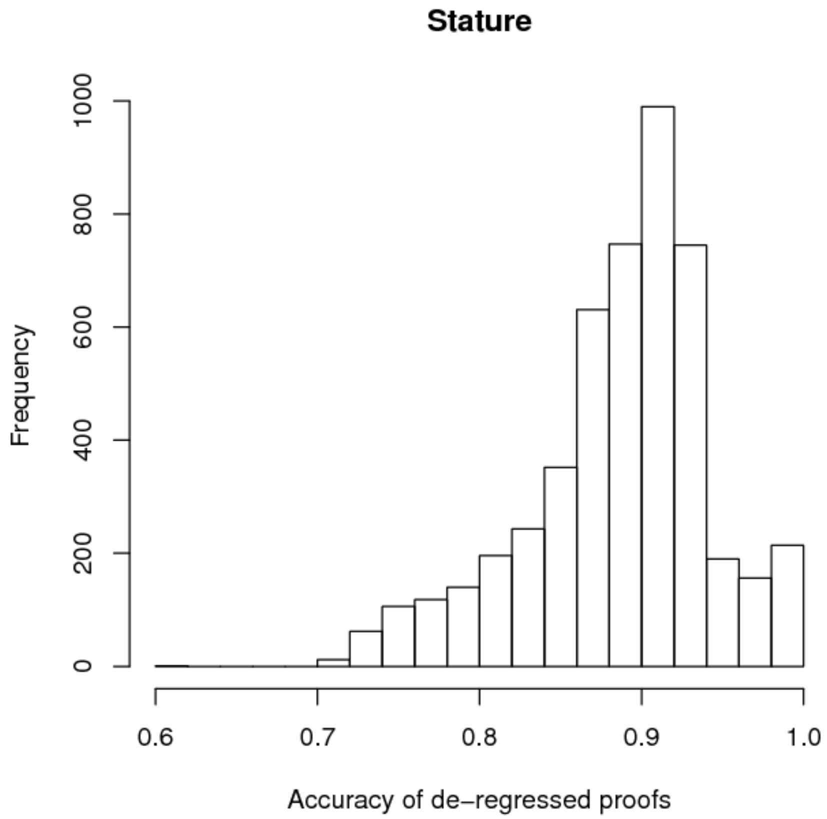

Table S1 Top twenty markers on chromosome 6 associated with service sire calving index (SCI) using a sire-model in Nordic Red cattle. Markers are arranged in descending order of –$\log_{10}$ (*P*-value)

SNP	rs-ID	Position (bp)	MAF	Effect	SE	–$\log_{10}$(p-value)	LD with Chr6:38127504	Variant type
Chr6:38127504	rs110329232	38127504	0.312	-3.769	0.231	57.502	1	5_prime_UTR_variant
Chr6:39697560	rs380475261	39697560	0.367	3.394	0.221	51.032	0.16	intergenic variant
Chr6:39685188	rs109068573	39685188	0.370	3.380	0.221	50.862	0.16	intergenic variant
Chr6:39683942	rs383383037	39683942	0.369	3.366	0.220	50.753	0.16	intergenic variant
Chr6:39687057	rs110152364	39687057	0.369	-3.367	0.220	50.747	0.16	intergenic variant
Chr6:38956499	rs109301655	38956499	0.084	-6.470	0.427	49.737	0.02	Intron variant
Chr6:39142464	NA	39142464	0.083	-6.460	0.427	49.629	0.02	intergenic variant
Chr6:39142325	rs109336064	39142325	0.083	-6.460	0.427	49.629	0.02	intergenic variant
Chr6:39142333	rs110116541	39142333	0.083	-6.460	0.427	49.629	0.02	intergenic variant
Chr6:39181207	NA	39181207	0.083	-6.459	0.427	49.629	0.02	intergenic variant
Chr6:39164044	rs379180947	39164044	0.083	-6.459	0.427	49.615	0.02	intergenic variant
Chr6:39165804	rs384077114	39165804	0.083	-6.459	0.427	49.615	0.02	intergenic variant
Chr6:39140538	rs208984737	39140538	0.083	-6.470	0.428	49.612	0.02	intergenic variant
Chr6:39142430	rs382938133	39142430	0.083	-6.459	0.427	49.612	0.02	intergenic variant
Chr6:39148421	rs110704385	39148421	0.083	-6.458	0.427	49.612	0.02	intergenic variant

Chr6:39139362	rs110186942	39139362	0.083	-6.458	0.427	49.611	0.02	intergenic variant
Chr6:39139659	NA	39139659	0.083	-6.458	0.427	49.611	0.02	intergenic variant
Chr6:39139764	rs384540439	39139764	0.083	-6.458	0.427	49.611	0.02	intergenic variant
Chr6:39105359	rs207689046	39105359	0.082	-6.575	0.435	49.609	0.02	intergenic variant
Chr6:39072546	rs109576691	39072546	0.083	-6.458	0.427	49.608	0.02	intergenic variant

rs-ID = reference SNP identification; MAF = minor allele frequency; effect = allele substitution effect for the SNP; SE = SE for the SNP effect; LD = linkage disequilibrium; NA = not available.

Table S2 Top twenty markers on chromosome 6 associated with body conformation index (BCI) using a sire-model in Nordic Red cattle. Markers are arranged in descending order of $-\log_{10}$ (*P*-value)

name	rs-ID	Position (bp)	MAF	Effect	SE	$-\log_{10}$(p-value)	LD with Chr6:39685188	Variant type
Chr6:39685188	rs109068573	39685188	0.396	-4.356	0.209	89.446	1	intergenic variant
Chr6:39697560	rs380475261	39697560	0.393	-4.363	0.210	89.367	1	intergenic variant
Chr6:39683942	rs383383037	39683942	0.395	-4.336	0.208	89.211	1	intergenic variant
Chr6:39687057	rs110152364	39687057	0.395	4.332	0.209	89.001	1	intergenic variant
Chr6:38127504	rs110329232	38127504	0.340	4.364	0.219	82.406	0.16	intergenic variant
Chr6:39750067	NA	39750067	0.448	-4.057	0.207	79.689	0.57	intergenic variant
Chr6:39750084	NA	39750084	0.450	-4.066	0.208	79.663	0.57	intergenic variant
Chr6:39743900	rs382209066	39743900	0.426	4.096	0.209	79.619	0.59	intergenic variant
Chr6:39742108	rs384995333	39742108	0.433	-4.056	0.207	79.544	0.59	intergenic variant
Chr6:39738116	rs385682668	39738116	0.442	-3.991	0.204	79.532	0.58	intergenic variant
Chr6:39743242	rs378728820	39743242	0.427	4.089	0.209	79.486	0.58	intergenic variant
Chr6:39736273	rs209355309	39736273	0.442	-3.990	0.204	79.460	0.59	intergenic variant
Chr6:39736193	rs207702574	39736193	0.442	-3.989	0.204	79.445	0.59	intergenic variant
Chr6:39738853	rs211509820	39738853	0.441	-3.984	0.204	79.364	0.59	intergenic variant

Chr6:39743937	rs379917426	39743937	0.431	4.047	0.207	79.314	0.59	intergenic variant
Chr6:39738209	rs383270383	39738209	0.440	-3.971	0.203	79.281	0.59	intergenic variant
Chr6:39743625	rs382359324	39743625	0.433	4.025	0.206	79.255	0.59	intergenic variant
Chr6:39740380	NA	39740380	0.440	-3.968	0.203	79.223	0.59	intergenic variant
Chr6:39736135	rs210654216	39736135	0.439	-3.967	0.203	79.220	0.59	intergenic variant
Chr6:39737297	rs110241699	39737297	0.440	-3.967	0.203	79.205	0.59	intergenic variant

rs-ID = reference SNP identification; MAF = minor allele frequency; effect = allele substitution effect for the SNP; SE = SE for the SNP effect; LD = linkage disequilibrium ; NA = not available.

Table S3 SNPs from the LCORL gene showing the most significant association with calf size (SCSL) used for constructing haplotypes (HAP1)

SNP	rs-ID	Position (bp)	MAF	Effect	SE	$-\log_{10}$(p-value)	Variant type
Chr6:38893987	rs385255004	38893987	0.083	6.43	0.286	105.928	intron variant
Chr6:38908960		38908960	0.079	6.751	0.300	105.788	intron variant
Chr6:38910101	rs379009029	38910101	0.079	6.751	0.300	105.785	intron variant
Chr6:38910119	rs381823183	38910119	0.079	6.751	0.300	105.788	intron variant
Chr6:38911548	rs109256415	38911548	0.083	6.426	0.285	105.821	intron variant
Chr6:38914196	rs110995268	38914196	0.083	6.425	0.285	105.821	intron variant
Chr6:38942103	rs109188585	38942103	0.083	6.416	0.285	105.813	intron variant
Chr6:38947564	rs110369727	38947564	0.083	6.408	0.285	105.818	intron variant
Chr6:38956109	rs109810741	38956109	0.084	6.414	0.285	105.802	intron variant
Chr6:38956499	rs109301655	38956499	0.084	6.415	0.285	105.992	intron variant
Chr6:38960730	rs109315734	38960730	0.083	6.408	0.285	105.825	intron variant

Chr6:38963718	rs377959514	38963718	0.083	6.409	0.285	105.847	intron variant
Chr6:38972255		38972255	0.084	6.408	0.285	105.675	intron variant
Chr6:38977050	rs109929997	38977050	0.083	6.408	0.285	105.824	intron variant
Chr6:38981742	rs109673676	38981742	0.082	6.502	0.289	105.826	intron variant

rs-ID = reference SNP identification; MAF = minor allele frequency; effect = allele substitution effect for the SNP; SE = SE for the SNP effect; NA = not available.

Table S4 SNP markers showing most significant association with body conformation used for constructing haplotypes (HAP2) and haplotype-based association analysis

Chromosome	rs_ID	Position (bp)	MAF	Effect	SE	$-\log_{10}$(p-value)	Variant type
Chr6:38700703	NA	38700703	0.217	5.110	0.253	85.071	intergenic variant
Chr6:38702123	NA	38702123	0.217	5.110	0.253	85.071	intergenic variant
Chr6:38703870	NA	38703870	0.217	5.110	0.253	85.071	intergenic variant
Chr6:38706518	NA	38706518	0.217	5.110	0.253	85.071	intergenic variant
Chr6:38707082	NA	38707082	0.217	5.110	0.253	85.071	intergenic variant
Chr6:38709292	NA	38709292	0.217	5.110	0.253	85.071	intergenic variant
Chr6:38714200	NA	38714200	0.217	5.110	0.253	85.071	intergenic variant
Chr6:38721008	NA	38721008	0.217	5.110	0.253	85.071	intergenic variant
Chr6:38734907	NA	38734907	0.217	5.110	0.253	85.071	intergenic variant
Chr6:38763321	NA	38763321	0.217	5.109	0.253	85.054	upstream gene variant
Chr6:38765427	NA	38765427	0.217	5.110	0.253	85.055	upstream gene variant
Chr6:38765668	NA	38765668	0.217	5.109	0.253	85.055	upstream gene variant
Chr6:38766261	NA	38766261	0.217	5.109	0.253	85.051	intron variant

Chr6:38810147	NA	38810147	0.218	5.118	0.253	85.271	intron variant
Chr6:38810462	NA	38810462	0.218	5.117	0.253	85.274	intron variant
Chr6:38914033	NA	38914033	0.220	5.165	0.252	87.201	intron variant
Chr6:38924562	NA	38924562	0.220	5.163	0.252	87.176	intron variant
Chr6:38934652	NA	38934652	0.220	5.163	0.252	87.176	intron variant
Chr6:38961998	NA	38961998	0.219	5.163	0.252	87.090	intron variant
Chr6:39008980	NA	39008980	0.219	5.163	0.252	87.097	intergenic variant
Chr6:39030068	NA	39030068	0.219	5.163	0.252	87.102	intergenic variant
Chr6:39052140	NA	39052140	0.219	5.168	0.252	87.141	intergenic variant
Chr6:39077951	NA	39077951	0.219	5.168	0.252	87.141	intergenic variant
Chr6:39078732	NA	39078732	0.219	5.168	0.252	87.141	intergenic variant
Chr6:39078733	NA	39078733	0.219	5.168	0.252	87.141	intergenic variant

rs-ID = reference SNP identification; MAF = minor allele frequency; effect = allele substitution effect for the SNP; SE = SE for the SNP effect; NA = not available.

Table S5 Model Comparisons for haplotype based association analyses, to include HAP1, HAP2, or both haplotypes in the model

Trait	M0 = breed + polygene		M1 = breed + HAP1 + polygene		M2 = breed + HAP2 + polygene		M3 = breed + HAP1 + HAP2 + polygene	
	ErrVar.M0	AIC.M0	ErrVar.M1	AIC.M1	ErrVar.M2	AIC.M2	ErrVar.M3	AIC.M3
Stature	6.13	20566.99	1.12E-04*	20415.55	11.28	20076.7	0.30	19863.92
Body Depth	9.13	18761.32	5.02	18735.04	11.72	18534.85	6.33	18496.18
Chest Width	7.16	19071.51	4.87	19024.37	10.17	18818.42	7.02	18750.14
Dairy Form	4.34	17806.93	4.19	17808.6	4.65	17675.5	4.41	17676.60
Rump Width	1.42	18357.25	3.00E-02*	18320.94	3.94	18069.05	2.68	18013.31
Rump Angle	8.31	18102.36	8.28	18105.7	8.44	17970.79	8.41	17974.03
SCEF	16.78	22047.29	10.13	21718.57	16.68	21585.39	9.88	21172.46
SCEL	26.78	23022.00	19.89	22674.6	26.01	22562.11	18.79	22130.79
SSBF	26.17	25805.45	11.12	25667.12	29.20	25350.37	13.38	25171.08

SSBL	15.05	25775.05	1.86E-04*	25653.54	19.54	25398.42	1.31	25247.92
SCSF	21.79	21110.13	17.71	20741.85	20.85	20694.2	16.33	20242.95
SCSL	20.66	20741.15	17.56	20291	19.97	20368.18	16.44	19829.67
SCI	35.87	25955.95	16.14	25778.34	38.28	25486.63	18.49	25259.49
BCI	9.60	17666.70	3.60	17537.62	13.94	17220.57	5.25	17026.02

* Model did not converge due numerical instability (very low error variance) and therefore could not estimate the variance component; ErrVar = Error variance, AIC = Akaike Information Criterion
